# CellVizio^®^ System for Mesothorax Lymphadenopathy Rapid on Site, 18G needle: Pros and Cons

**DOI:** 10.7150/jca.136122

**Published:** 2026-05-25

**Authors:** Paul Zarogoulidis, Wolfgang Hofenforst-Schwidt, Haidong Huang, Stavros Tryfon, Yifei Zhang, Guoren Ma, Kyriakos Amarantidis, Elena Maragouli, Vasilis Papadopoulos, Eleni-Isidora Perdikouri, Vagelis Voulgaris, Lutz Freitag, Chrysoula Margioula-Siarkou, Theodora Tsiouda, Paschalis Ntolios, Stamatis Petousis, Konstantina Triantafylopoulou, Kosmas Tsakiridis, Nikolaos Courcoutsakis, Sofia Baka, Dimitris Baloukas, Paschalis Steiropoulos, Bojan Zaric, Xiaping Shen

**Affiliations:** 1 Pulmonary-Oncology Department, General Clinic Imithea, Thessaloniki, Greece.; 2 Thorax Centre Südwestfalen, Märkische Kliniken, ''Lüdenscheid'' Clinics, aff. University of Bonn and Private University of Hamburg, Germany.; 3 Department of Respiratory and Critical Care Medicine, The First Affiliated Hospital of Naval Medical University (Shanghai Changhai hospital), Shanghai, China.; 4 Pulmonary-Oncology Department, 'G.Papanikolaou' General Hospital, Thessaloniki, Greece.; 5 Department of Radiology, The First Affiliated Hospital of Naval Medical University (Shanghai Changhai hospital), Shanghai, China.; 6 The Fourth People's Hospital of Ningxia Hui Autonomous Region, Yinchuan, China.; 7 Oncology Department, General University Hospital of Alexandroupolis, Democritus University of Thrace, Alexandroupolis, Greece.; 8 Oncology Department, General University Hospital of Thessaly, University of Thessaly, Larissa, Greece.; 9 Oncology Department, General Hospital of Volos, Volos, Greece.; 10 Division of Interventional Pneumology, Department of Pulmonary Medicine, University Hospital Essen - Ruhrlandklinik, Essen, Germany.; 11 Second Department of Obsetrics and Gynaecology, Aristotle University of Thessaloniki, Thessaloniki, Greece.; 12 Pulmonary-Oncology Department, Theageneio Cancer Center, Thessaloniki, Greece.; 13 Internal Medicine, Nursing School, Democritus University of Thrace, General University Hospital of Alexandroupolis, Thrace, Greece.; 14 Thoracic Surgery Department, Interbalkan Medical Center, Thessaloniki, Greece.; 15 Radiology Department, General University Hospital of Alexandroupolis, Democritus University of Thrace, Alexandroupolis, Greece.; 16 Oncology Department, 'G. Papageorgiou' General University Hospital, Aristotle University of Thessaloniki, Thessaloniki, Greece.; 17 Pulmonary Department, University General Hospital of Alexandroupolis, Democritus University of Thrace, Alexandroupolis, Greece.; 18 Faculty of Medicine, University of Novi Sad, Institute for pulmonary diseases of Vojvodina, Serbia.; 19 Department of Radiology, The First Affiliated Hospital of Naval Medical University (Shanghai Changhai hospital), Shanghai, China.

**Keywords:** bronchoscopy, ebus, confocal microscopy, Cellvizio^®^, Pentax, rose, 18G needle

## Abstract

**Introduction:**

The best tissue sample is still very important for the diagnosis of mesothorax lymphadenopathy. In the past 20 years endobronchial ultrasound (EBUS) has been used efficiently in most cases of primary lung cancer disease or metastatic disease. Several new type of needles have been created such 19G, 18G and hybrid biopsies with cryoprobes. Rapid on-site evaluation (ROSE) is used as an additional initial diagnostic tool. Confocal microscopy is a method of rapid on-site evaluation.

**Patients and Methods:**

One hundred patients with mesothorax lymphadenopathy were biopsied with ebus and two groups were created one with rapid on-site evaluation with confocal microscopy and one by an operator with microscopic evaluation with sample preparation on cytoglasses. Our main objective was to assess parameters such as time, false negative results between the two techniques and technical issues such as the accessibility and evaluation between the two techniques. Safety was also evaluated.

**Results:**

Rapid on-site evaluation is more cost efficient with a cytologist on site, however; with a higher rate of false negative results. Accessibility with the Cellvizio^®^ catheter was less possible in a few cases due to rigid angles within the airways especially for small lymphnodes ≤ 1.5 cm (L4L mainly).

**Discussion:**

Confocal is a safe and time-efficient technique for mesothorax lymphadenopathy. A high level of training is required for pulmonary physicians in order to assess the novel imaging technique. Definitely different parts of the lymphnode have to reached and evaluated in order to have a proper initial evaluation.

## Introduction

Computed tomography of the thorax with low dose radiation has been used in the past 5 years as a method of early lung cancer diagnosis. 1 However; there was still an issue with the biopsy of mesothorax lympnodes. Enlarged lymphnodes of the mesothorax could be attributed to a primary lung cancer such as; non-small cell lung cancer (NSCLC), etc. small cell lung cancer (SCLC), metastasis from a previous primary cancer such as; breast cancer, colon cancer or ovarian cancer. Moreover; it could be due to infection such as; tuberculosis or connective tissue disease-sarcoidosis. 2, 3 In the past 18 years advanced bronchoscopic techniques have been included in the diagnosis. Endobronchial ultrasound endoscopes have been used for more than decade.^4^ Before the advanced bronchoscopic techniques we used to perform biopsy with mesothoracoscopy. 5 Positron emission tomography added valuable information regarding the optimal site for biopsy and the status of the lymphnodes whether they are active or not. 6 Moreover; we tried additional techniques such as; elastography to visualize the optimal site for biopsy within a lymphnode, without any positive results that could be incorporated in the everyday clinical practice. 7, 8 Additionally, to all these biopsy methods another diagnostic tool was added in the process; rapid on site evaluation (ROSE). It provides the ability to check a sample whether it is sufficient for diagnosis and also if we have cancer cells. 9 The next step of evolution for rapid on-site evaluation was the creation of confocal microscopy with the CellVizio^®^ system. 10 This system provides a real-time assessment during the biopsy procedure of the tissue that we are investigating and therefore we choose the best site of biopsy. We definitely increase the safety of the biopsy site procedure since we perform less biopsies and we enhance our performance since we choose the best site for biopsy. Moreover; we also have a clear image whether we have cancer or not. Confocal microscopy- CellVizio^®^ has been included in several procedures with robotic assistance or conventional guidance with fluoroscopy systems. It has also been used in other specialties such as gastroenterology. 11 The cost still remains an issue, however; this technology will outrun rapid on-site evaluation in the clinical setting. Artificial intelligence is the next step for confocal microscopy where the system itself will indicate the cancer cells while performing the scanning. In our study we investigated the safety and efficiency of rapid on-site evaluation in comparison to confocal miscroscopy during sampling of mesothorax lymphadenopathy.

## Patients and Methods

One hundred patients with lymphadenopathy ≥ 1.5 cm were divided in two groups of 50 patients. Fifty patients were investigated with Pentax EB-1970UK endoscope and confocal microscopy. CLE probe AQ-Flex^TM^ (Mauna Kea) through an 18G needle Broncus Flex needle. The other fifty again the same procedure however; instead of confocal microscopy we used traditional rapid on-site evaluation (ROSE) with preparation of the material on cytoglasses and an Olympus microscope. All patients included underwent the bronchoscopic procedure with mild sedation and jet-ventilation. All procedures were performed from 8-15 days from last computed tomography scan. Possitron emission tomography was also performed in all patients and only patients with SUV ≥ 3 were included in the study (5-7days before the study). The group with confocal microscopy (CLE) was named 1 and group with TROSE 2. Moreover; we had both groups subdivided in two groups with lymphnodes up to 1.5-2 cm and 2 > cm. Patients with lymphnodes ≤ 1.5cm were excluded from the study. All patients were ≥ 18 years old fit to undergo bronchoscopy and in total 78 males and 22 females participated. In group 1 twenty patients had lymphnodes ≤ 2 cm and thirty > 3 cm, while in group 2 sixteen patients had lymphnodes ≤ 2 cm and thirty-four > 2 cm. Four patients had false negative results and three were negative for cancer. In group 2 we had twelve false negative results and again three were negative for cancer. Fluocyne 10% (5 ml/10 ml) was injected in group 1 while the procedure was ongoing. Fluorescein is the most commonly used agent. The fluorescein sodium is a Food and Drug Administration (FDA) class IIa drug which has been approved by the FDA for ophthalmic angiography or angioscopy of the retina and iris vasculature in conjunction with a confocal scanning laser ophthalmoscope. The fluorescein monoglucuronide has fluorescent properties and contributes about 20% of fluorescence as compared to unbound fluorescein. After IV administration, the urine remains slightly fluorescent for 24 to 36 hours. A renal clearance of 1.75 mL/min/kg and a hepatic clearance (due to conjugation) of 1.50 mL/min/kg have been estimated. The systemic clearance of fluorescein was essentially complete by 48 to 72 hours after administration of 500 mg fluorescein. The safety of fluorescein sodium in gastrointestinal CLE has been investigated recently. A large multi-center study led by our group including 2272 patients (excluding pregnant and breast-feeding females), evaluated the safety of intravenous use of 2.5 mL to 5 mL of 10% fluorescein sodium. This study showed very low rates of mild (1.4%) and serious (0%) side effects during immediate post procedure period. In addition to its good safety profile and rare side effects, fluorescein is inexpensive, easy to use, and has excellent fluorescent properties. 12 The optimal dose of fluorescein for high quality pCLE imaging has been previously investigated and it is approximately 5.0 mL. 13 In group 1 we had two mild bleeding events and had to be hospitalized for two days max. In group 2 we had four patients with mild bleeding and again hospitalization was maximum two days. **Table 1.** In group 1 nine patients were previously diagnosed with prostate cancer, two with thyroid cancer, three with breast cancer or four with colon cancer. In group 2 five patients were previously diagnosed with prostate cancer, one with thyroid, four with breast cancer and three with colon cancer. **Table 2.** Moreover; in group 1 three patients had previously diagnosed with rheumatoid arthritis and one with scleroderma and were under biological treatment. On group 2 four patients had previously diagnosed with rheumatoid arthritis and one with scleroderma and were under biological treatment. We included in our study patients only with lymphadenopathy and in 55 cases we performed biopsy from 2 lymphnode stations. The parameter time was evaluated as follows: for group 1; timer started when the confocal catheter probe punctured the lymphnode and evaluation by the operator finished. Again, for group 2; timer started when the biopsy sample was put on cytoglasses and microscope. We had two 'time' groups E = 2 min and F = >2 min. Patients were released the same day after 2 hours of the procedure. Only 6 patients remained hospitilised with mild bleeding up to 2 days max.

### Equipment

**Figure 1:**
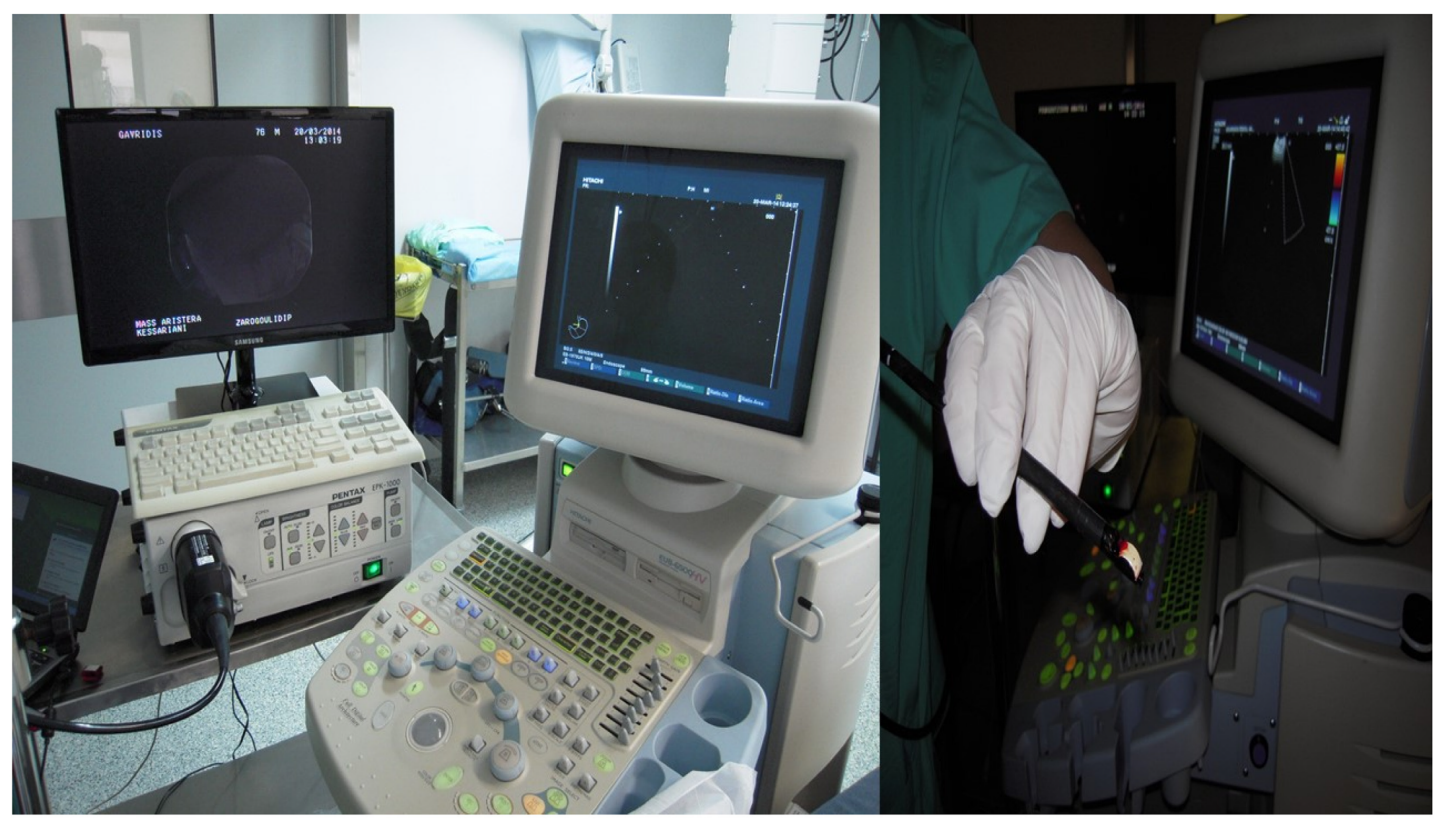
Pentax ebus system EB-1970UK.

**Figure 2:**
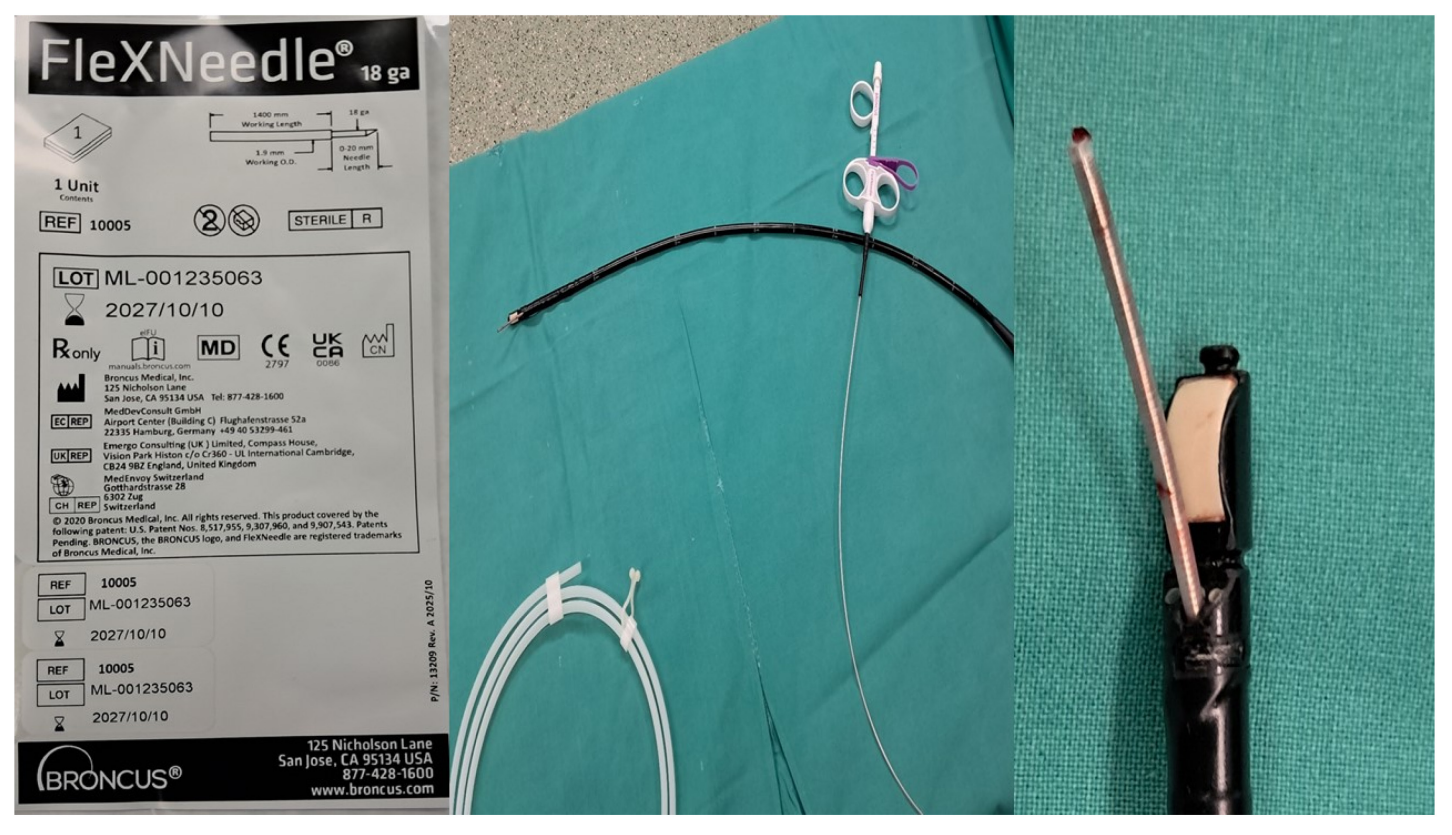
18G needle Flex from Broncus^®^, San Jose, CA 95134, U.S.A. inserted through the working channel of an EB-1970UK Pentax Endoscope.

**Figure 3:**
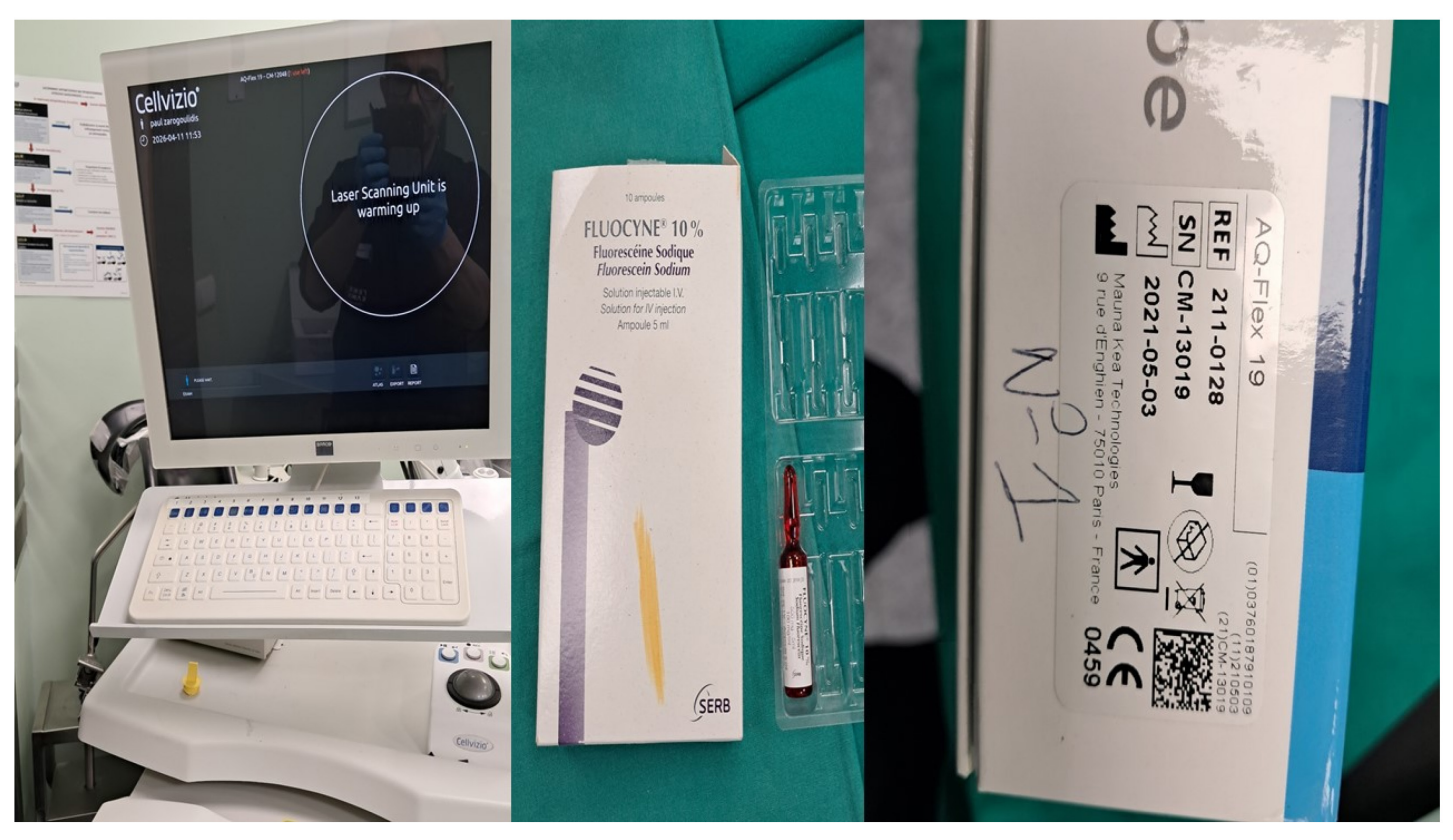
Cellvizio® confocal microscopy system 488nm, Mauna Kea Technologies, with AQ-Flex^™^ 19 (-,IR) N probe, Remma suatained Healthcare.

**Figure 4:**
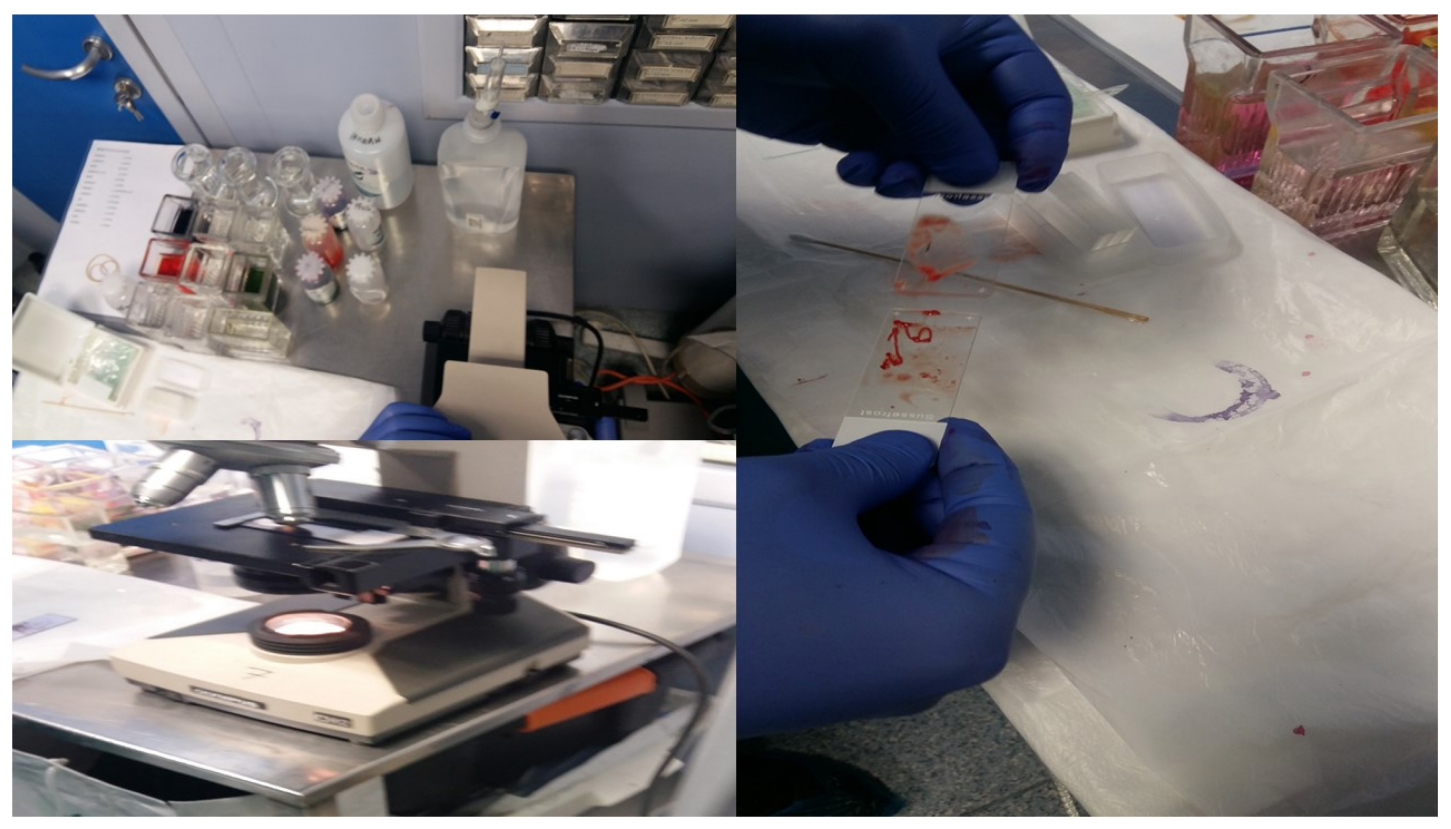
Rapid on site evaluation preparation solutions and microscope.

**Figure 5:**
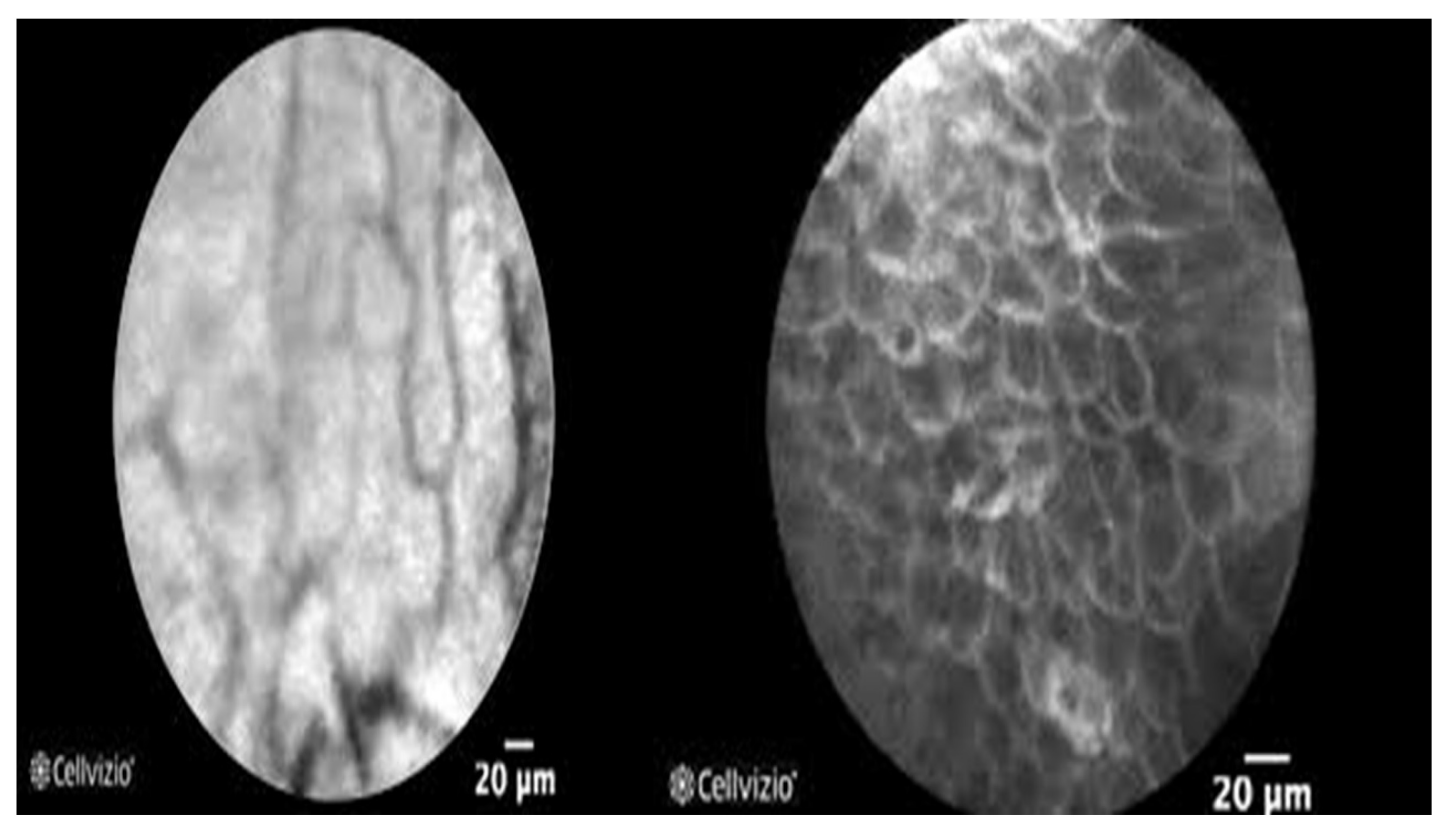
CLE images, left necrosis and right adenocarcinoma.

We used Pentax EB-1970UK video-bronchoscope with 2.8mm working channel, Papapostolou S.A, Greece (**Figure 1**.), 18G needle Flex from Broncus^®^, San Jose, CA 95134, U.S.A. (**Figure 2**.) , Cellvizio® confocal microscopy system 488nm, Mauna Kea Technologies, with AQ-Flex^™^ 19 (-,IR) N probe, Remma suatained Healthcare (**Figure 3**.), Rapid on site evaluation was performed by the operator (**Figure 4**.) and CLE was evaluated again by an experienced operator (Paul Zarogoulidis, Wolfgang Hohenforst-Schmidt, haidong Huang and Bojan Jaric) (**Figure 5**).

## Results

**Figure 7:**
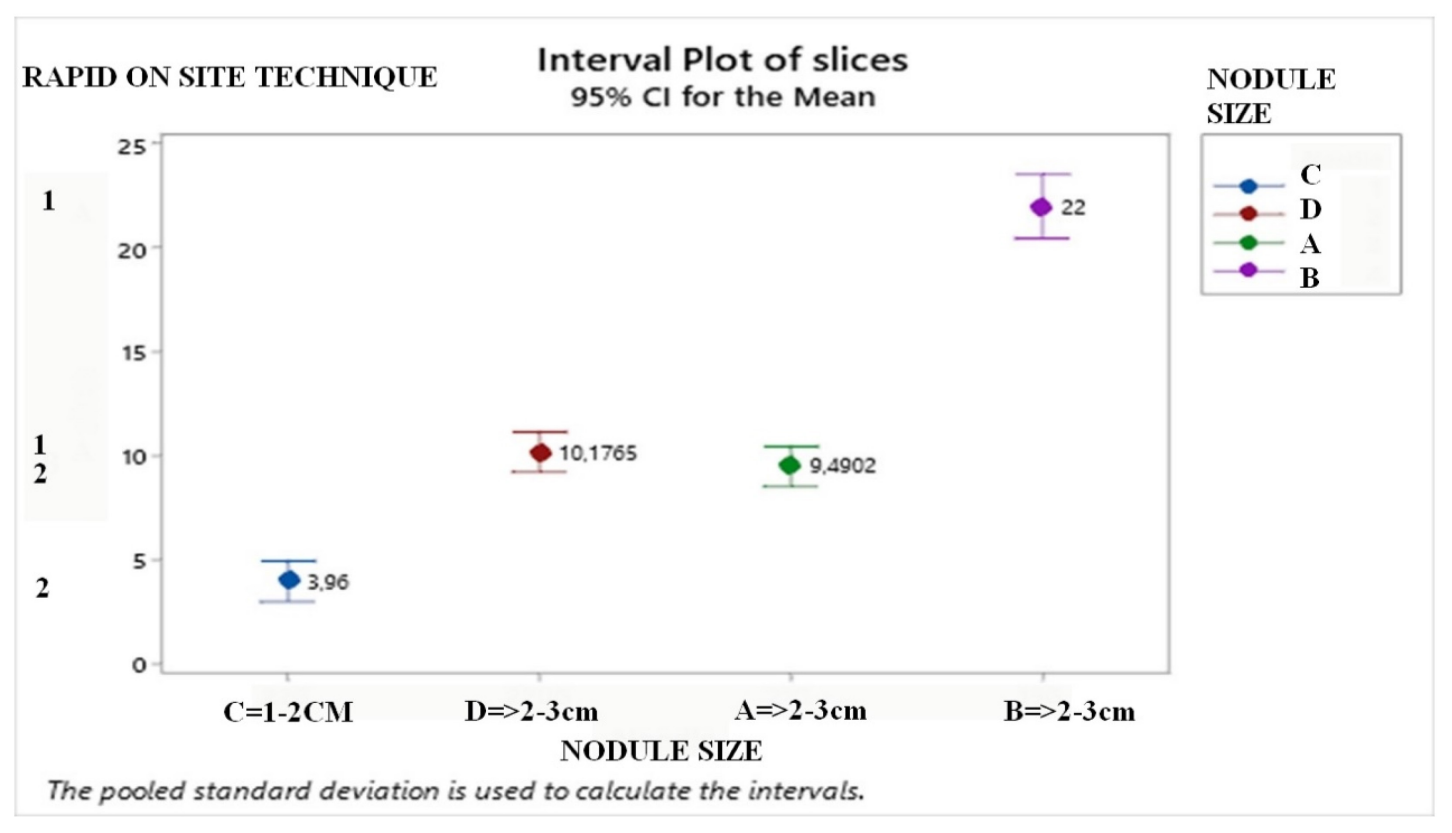
False negatives according to the type of rapid on-site evaluation and nodule size. Vertical lines denote the 95% confidence intervals of means based on the ANOVA's error mean square. Levels means whose intervals do not overall differ significantly.

For confocal microscopy less false negative results were observed. Moreover; less false negative results were observed when lyphnodes were >2cm in size and when > 2 stations were punctured. Almost the same false negative results were observed between confocal microscopy and TROSE group 2 when the lymphnodes ≤ 2 cm and only one station was biopsied. Regarding TROSE group 2false negative results were observed almost the same between the different lyphnode size and multiple stations biopsy, however; with less observed when the lymphnode size was > 2 cm. **Figure 7**.

**Figure 8:**
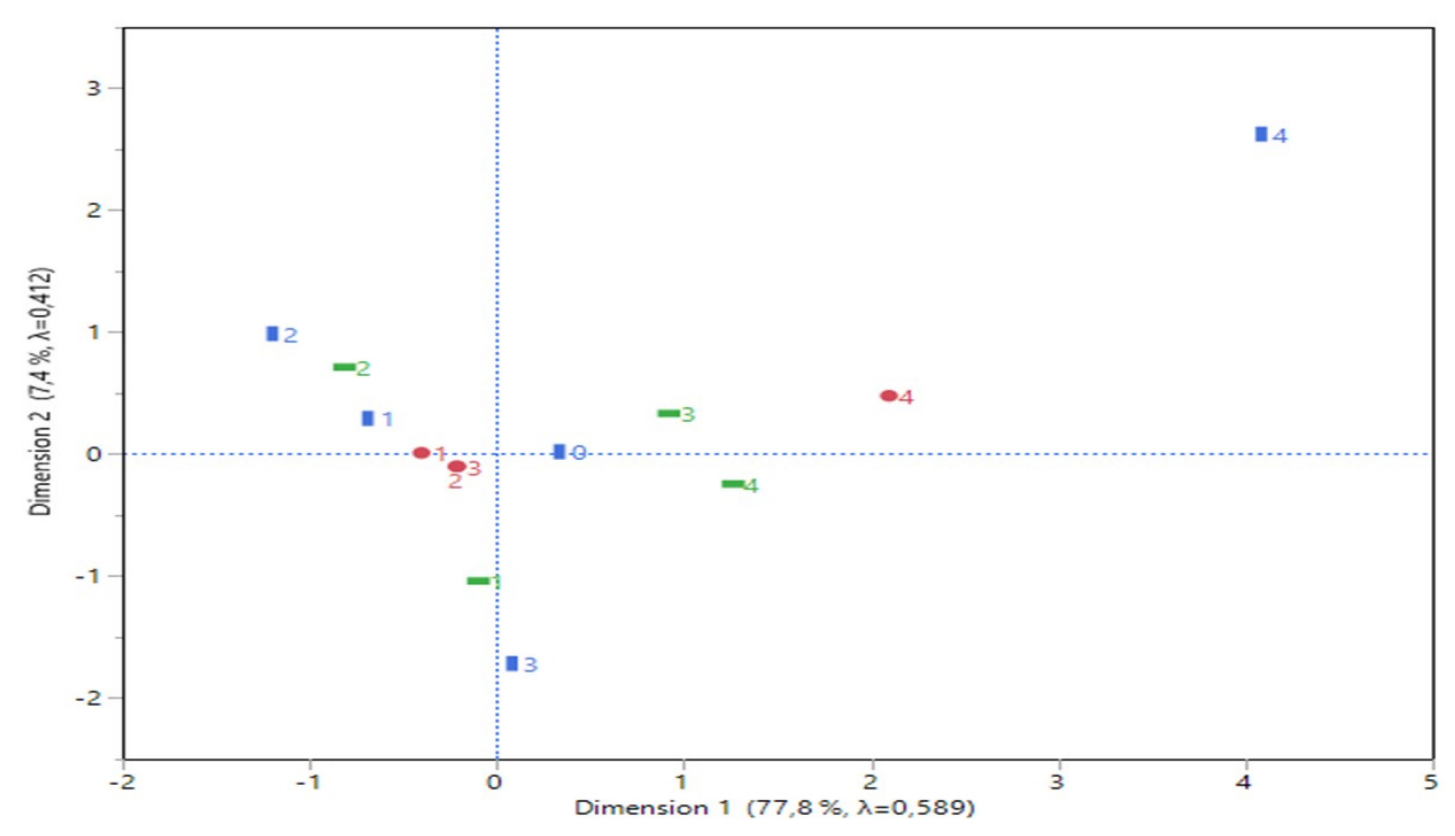
Statistical output of a correspondence analysis: a two dimensions plot, the Greenacre adjusted inertia (percentage contribution to the first two dimensions) and partial contributions of coordinates to each dimension.

The multiple correspondence analysis pointed out an 85,25% explanation of inertia variation (**Figure 8.**) concerning the first two dimensions.

Dimension 1 is best described by confocal microscopy lymphnode size > 2 cm and 2 stations, both arranged at the right part of corresponding plot and far apart from the rest categories. Similarly, dimension 2 is best formed by lymphnode size group 1 (< 2 cm) and Cellvizio®, both positioned at the lower part of the plot and also far apart from the other categories. Rapid on-site evaluation from an operator for pulmonary nodules > 2 cm are also affiliated each other due to their close distance and moderate contributions for the axes formation. There were less false negatives for the operator group when the lymphnode size was > 2 cm and 2 stations were sampled.

**Figure 9:**
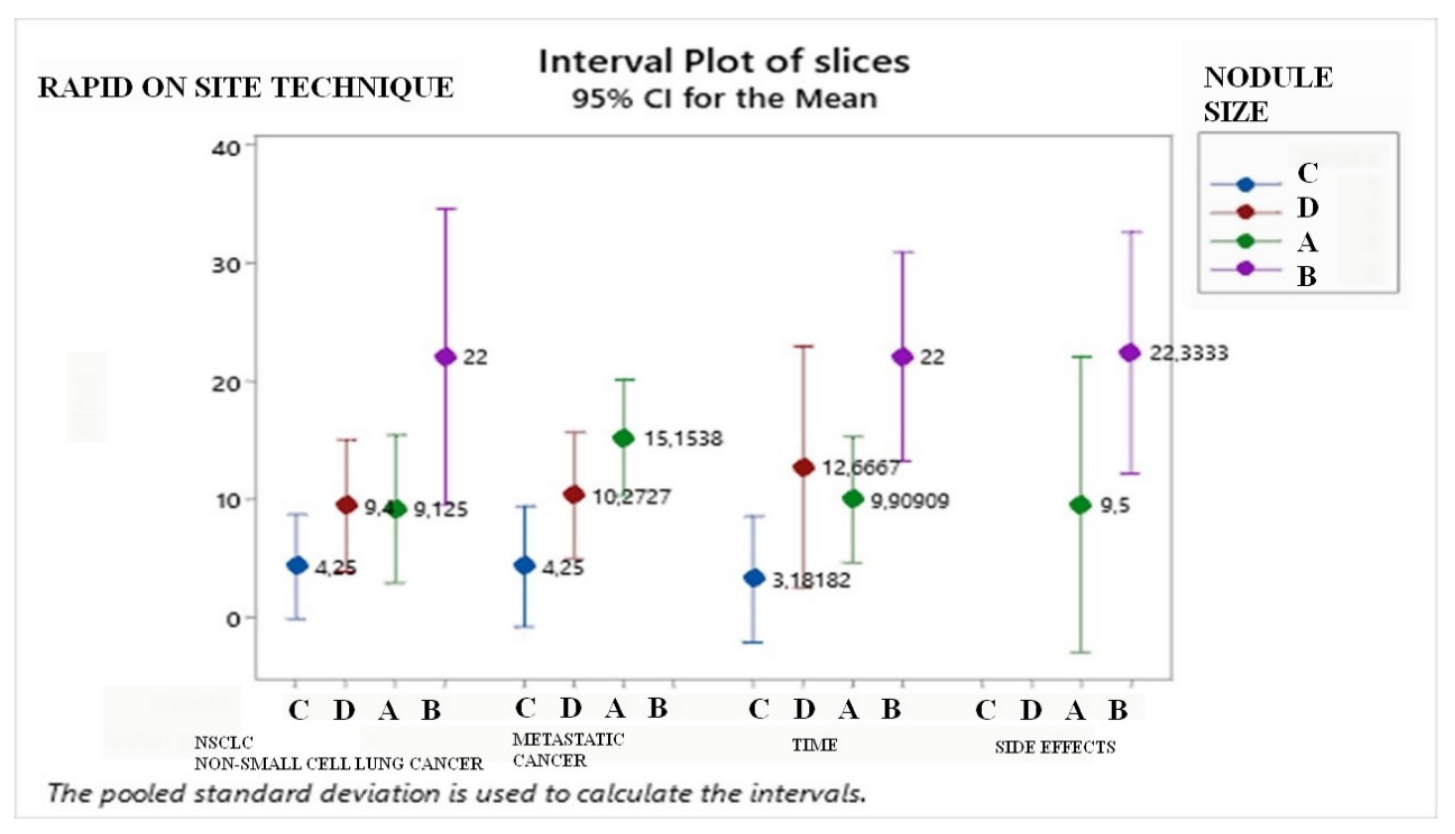
Mean number of false negatives distribution according to the combined effect of lymphnode size/number of sample stations and rapid on-site technique-group 2. Vertical lines denote the 95% confidence intervals of means based on the ANOVA's error mean square. Levels means whose intervals do not overall differ significantly.

Adenocarcinoma is easier to diagnose with confocal microscopy and lymphnodes > 2 cm in most cases in ≤ 2 minutes. Metastatic adenocarcinoma was also easier to diagnose with confocal microscopy in ≤ 2 cm. *95% confidence* interval plot (**Figure 9.**) indicate that confocal microscopy and lymphnode size > 2 cm, > 2 sample stations and primary adenocarcinoma or metastatic are close related, confidence interval weakness when with TROSE group 2, carcinoid, thyroid metastatic cancer and pulmonary nodule size ≤ 2 cm, connective tissue disease and time. Five patients were diagnosed with lymphoma, three with Non-hoghkin B-line and two with T-line. Two false negative lymphoma patients were in group 2. The false negative results were associated with group 2 (TROSE technique) lympnodes < 2 cm and less than 1 station sampled.

Confocal microscopy, lymphnode size > 2 cm and 2 station sampling, time and adenocarcinoma in fact present a proportional result due to the following reasons:

-The larger the lymphnode size > 2 cm and more stations sampled, the less false negative results.

-The cancer type adenocarcinoma > squamous cell carcinoma > carcinoid decreases the false negative results.

-The metastatic cancer type colon cancer > prostate cancer > thyroid cancer, decreases the false negative results.

-Connective tissue disease versus cancer, in order to be 100% only follow up will verify a negative result in connective tissue disease patients.

-Concerning the time of evaluation: confocal microscopy group 1 had less false negative results, indifferent of cancer type (including metastasis), or connective tissue disease. Side effects where not correlated with time, cancer type (including metastasis) or lymphnode size.

-Time of evaluation was not correlated with false negative results.

Nine patients were cancer free. Three in group 1 and three in group 2 confirmed later with computed tomography scan after. Three of them had connective tissue disease. However; six patients had mild bleeding two in group 1 and four in group 2 and had to be hospitalized for 2 days max.

## Discussion

Endobronchial ultrasound has proven an efficient biopsy technique with very high diagnostic effectiveness ≥ 95% when all information for a patient is combined; positron emission tomography scan and patient medical clinical history. 6, 14 Issues to be considered, firstly the cost-effect. Cellvizio^®^ technology is still expensive since the AQ-Flex probes can be used for up to 10 patients each and then a new one has to be purchased (8.500 euros per piece in our country). The system itself is expensive the newest model is ≥ 100.000euros and is available in most countries. Moreover; the probe is inserted through an 18G Flex-Broncus^™^ needle which again in our country is available for 1300euros a piece-single use. The price for a single use 22G needle (mediglobe which we use for Pentax) is 270euro with tax, 21G needle 360 euro and for a 19G Olympus needle which we can use with adapter in Pentax endoscopes is 770 euro with tax. Previous studies have proved that in most cases of lymphadenopathy 22G needles and cell blocks can provide initial diagnosis and molecular profiling. 14 The main diagnostic issue occurs when other malignancies such lymphomas, sarcoma, bronchial cyst, infection, thymoma, sarcoidosis or post chemotherapy lymphadenopathy and infectious lymphadenopathy occurs. Safety issues have been raised between 18G and 19G needles versus 21G, 22G and cryoprobes, however; the operator should consider the diagnostic value of each needle. Larger needles are chosen when lymphoma or thymoma are suspected. We have also several tools for hemostasis like polymer powder, argon plasma or balloon dilator. 15-18 Rapid on-site evaluation (TROSE) with the conventional method we need a microscope (600-6000euros) and liquid deeps, cytoglasses and a cytologist or certified pulmonary physician. The cost for TROSE per patient is less than 120euro at least for our department, in comparison to CLE which is almost 1300euro per patient. Cryo probe is 440euros and be used multiple times, the cryo ERBE II system is almost 20.000euros in our country. Second issue is safety; larger needles or cryoprobes can induce larger damage during biopsy and therefore additional preparation is needed. Spirometry or sleep test should be performed for some patients in case of intubation. Jet-ventilation with sedation should be considered for most patients especially when multiple biopsies from different lymph node stations is needed. Our study is a small study with only 100 patients included and with a strict population included in order to evaluate specifically the effectiveness of CLE versus TROSE by an experienced operator for mediastinal lymphnodes. Based on our results, CLE is an efficient diagnostic tool making a biopsy procedure shorter, safer, however; it could be more often used for other medical procedures, such as solitary pulmonary nodules. Solitary pulmonary nodules can be accessed with several diagnostic tools and CLE could be more efficient than TROSE, especially for those patients with severe emphysema where we need to keep local lung tissue damage to minimum.

## Ethics Approval

Ethical approval for this study was obtained and it is available upon request from the corresponding authors Dr. Paul Zarogoulidis and Dr, Haidong Huang.

## Informed Consent

Written informed consent was obtained from all patients upon admission in the clinic to perform the biopsy.

## Figures and Tables

**Figure 6:**
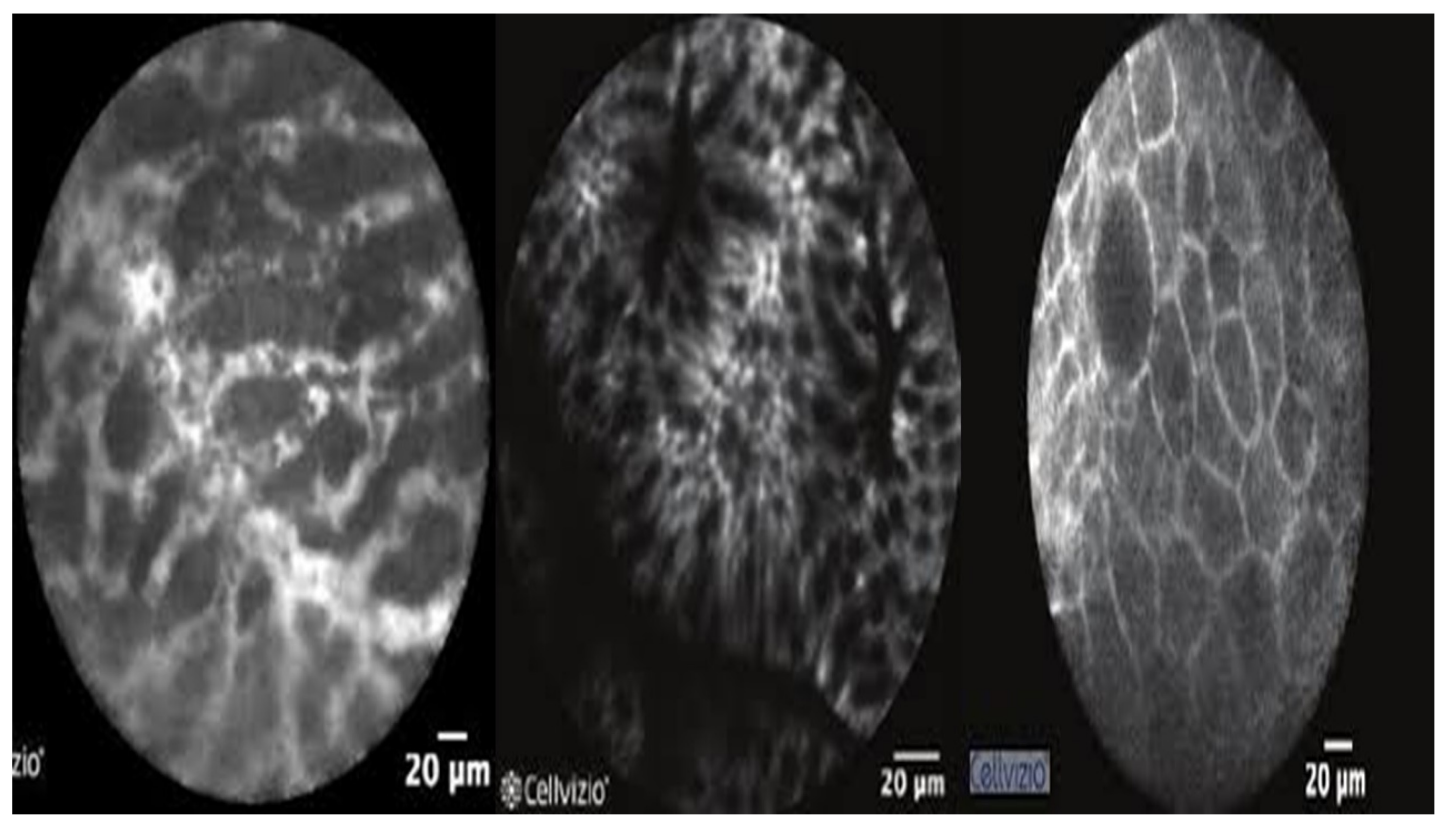
Left normal bronchial wall, middle normal lymphnode, right infiltrated cancerous lymphnode

**Table 1:** Study data 1

No.	Sex		Lymph node size		Metastasis		Connective tissue disease	Time	
	Male	Female	1- ≤ 2 cm	2-3 cm	Thyroid, Colon, Breast, Prostate		Rheumatoid arthritis, scleroderma	2 min	2 > min
Group 1	38	12	30	20	14	4	4	22	28
Group 2	40	10	34	16	8	4	5	14	36

**SPN:** Solitary Pulmonary Nodule, NSCLC: Non-Small Cell Lung Cancer

**Table 2:** Study data 2

No.	NSCLC diagnosis			Lymphoma	False negative	Prostate cancer	Breast cancer	Colon cancer	Thyroid cancer
	Adeno-carcinoma	Squamous cell	Neuro-endocrine						
Group 1	17	8	2	2	12	9	3	4	2
Group 2	18	13	1	3	4	5	4	3	1
False Negative	7	8	3	2		0	0	0	2

**Group 1** three patients negative for cancer upon follow-up **Group 2** three patients negative for cancer upon follow-up **NSCLC:** Non-small lung cancer **Lymphoma:** Non-Hodgin (3 B line and 2 T line)

## References

[1] Long KJ, Ward R, Kazerooni E, Pinsky P, Young R, Silvestri G (2026). New and growing nodules are strongly associated with malignancy in follow-up screens for lung cancer: a cohort study. Chest.

[2] Zajac P, Zajac M, Kadziolka W, Sokolowski A, Kaznowska E (2026). Diagnostic Factors Associated with Sarcoidosis in Patients Referred for EBUS-TBNA Due to Mediastinal Lymphadenopathy. Advances in respiratory medicine.

[3] Muchahary J, Nongpiur VN, Lyngdoh WV, Kalita P, Marbaniang E, Raphael V (2026). Diagnostic yield of endobronchial ultrasound-guided transbronchial needle aspiration for isolated mediastinal lymphadenopathy in a tuberculosis-endemic region: first study from Northeast India. Monaldi archives for chest disease = Archivio Monaldi per le malattie del torace.

[4] Oezkan F, Khan A, Zarogoulidis P, Hohenforst-Schmidt W, Theegarten D, Yasufuku K (2014). Efficient utilization of EBUS-TBNA samples for both diagnosis and molecular analyses. OncoTargets and therapy.

[5] Haruki T, Yamamoto H, Hoshikawa Y, Iwata H, Sato Y, Suzuki K (2025). Clinicopathological features and perioperative outcomes of robot-assisted thoracoscopic surgery for primary lung cancer: An analysis of initial outcomes based on the National Clinical Database. Surgery today.

[6] Zarogoulidis P, Huang H, Hu Z, Wu N, Wang J, Petridis D (2021). Priority of PET-CT vs CT Thorax for EBUS-TBNA 22G vs 19G: Mesothorax Lymphadenopathy. Journal of Cancer.

[7] Zarogoulidis P, Kosmidis C, Fyntanidou V, Barmpas A, Koulouris C, Aidoni Z (2019). Elastography during convex-probe (endobronchial ultrasound) for optimal biopsy sample and gene identification in non-small-cell lung cancer. Biomarkers in medicine.

[8] Huang H, Huang Z, Wang Q, Wang X, Dong Y, Zhang W (2017). Effectiveness of the Benign and Malignant Diagnosis of Mediastinal and Hilar Lymph Nodes by Endobronchial Ultrasound Elastography. Journal of Cancer.

[9] Ali MS, Dalsania N, Thomas N, Jewani S, Mehrotra S, Nasar A (2026). Diagnostic accuracy of rapid on-site evaluation (ROSE) during robotic bronchoscopy. Journal of thoracic disease.

[10] van Heumen S, Kramer T, Korevaar DA, Gompelmann D, Bal C, Hetzel J (2024). Bronchoscopy with and without needle-based confocal laser endomicroscopy for peripheral lung nodule diagnosis: protocol for a multicentre randomised controlled trial (CLEVER trial). BMJ open.

[11] Tahara T, Horiguchi N, Terada T, Yamada H, Yoshida D, Okubo M (2019). Diagnostic utility of probe-based confocal laser endomicroscopy in superficial non-ampullary duodenal epithelial tumors. Endoscopy international open.

[12] Wallace MB, Meining A, Canto MI, Fockens P, Miehlke S, Roesch T (2010). The safety of intravenous fluorescein for confocal laser endomicroscopy in the gastrointestinal tract. Alimentary pharmacology & therapeutics.

[13] Shahid MW, Crook JE, Meining A, Perchant A, Buchner A, Gomez V (2011). Exploring the optimal fluorescein dose in probe-based confocal laser endomicroscopy for colonic imaging. Journal of interventional gastroenterology.

[14] Notarangelo S, Lombardi M, Lombardi R, Liguori G, Sperandeo M, Ruga S (2026). Endobronchial ultrasound-guided transbronchial fine-needle aspiration of mediastinal lymph nodes: a clinical appraisal of diagnostic accuracy and molecular utility. Current medical research and opinion.

[15] Yabe H, Nishimaki T, Hirabuki S, Tsuchimoto J, Murao K, Aisaka H (2026). Rapidly Progressive Primary Pulmonary Leiomyosarcoma Diagnosed by Endobronchial Ultrasound-Guided Transbronchial Needle Aspiration: A Case Report. Respirology case reports.

[16] Zarogoulidis P, Huang H, Zhou J, Ning Y, Yang M, Wang J (2021). Thyroid cancer diagnosis with transdermal probe 22G U/S versus EBUS-convex probe TBNA-B 22G and 19G: pros and cons. Expert review of medical devices.

[17] Balaji KV, Chauhan NK, Dutt N, Aggarwal D (2025). Echoes of infection: radial EBUS unveils invasive pulmonary aspergillosis. BMJ case reports.

[18] Kwiatkowski R, Zielinski M, Paluch J, Gabor J, Swinarew A (2024). Enhancing Patient Selection in Stage IIIA-IIIB NSCLC: Invasive Lymph Node Restaging after Neoadjuvant Therapy. Journal of clinical medicine.

